# Efficacy and safety of modified laparoscopic single-position radical nephroureterectomy with bladder cuff resection for upper urinary tract urothelial carcinoma: A single-center retrospective study

**DOI:** 10.14440/bladder.2025.0007

**Published:** 2025-07-03

**Authors:** Fei Wu, Zhen Wang, Liang Sun, Meixia Zhang, Hao Ning, Zhihong Niu, Jiaju Lyu, Dexuan Gao

**Affiliations:** 1 Department of Urology, Shandong Provincial Hospital Affiliated to Shandong First Medical University, Jinan, Shandong 250014, China; 2 Department of Urology, Jinan Third People’s Hospital, Jinan, Shandong 250101, China; 3 Department of Urology, The Second Hospital of Shandong University, Jinan, Shandong 250199, China

**Keywords:** Upper urinary tract urothelial carcinoma, Radical nephroureterectomy, Modified technique, Bladder cuff resection

## Abstract

**Background:**

Radical nephroureterectomy (RNU) with bladder cuff excision represents the standard treatment for high-risk upper tract urothelial carcinoma (UTUC).

**Objective:**

This study aimed to evaluate the safety, feasibility, and clinical outcomes of a modified laparoscopic single-position RNU plus bladder cuff resection.

**Methods:**

This retrospective analysis examined patients diagnosed with UTUC who underwent RNU between May 2022 and July 2024. Participants were divided into three groups: Group A (39 patients) underwent the modified technique, Group B (38 patients) received standard laparoscopic nephroureterectomy with bladder cuff resection, and Group C (27 patients) had laparoscopic nephroureterectomy with an additional lower abdominal incision for bladder cuff resection. We compared baseline characteristics, intraoperative variables, and post-operative outcomes among the groups.

**Results:**

A total of 104 patients were included and analyzed. Their baseline characteristics showed no significant differences among groups (*p*>0.05). Group A experienced a significantly shorter operative time and earlier ureteral catheter removal compared to Groups B and C (*p*<0.05). Intraoperative blood loss, gastrointestinal recovery time, and length of hospital stay were comparable between Groups A and B, but the results in the two groups were more favorable against Group C (*p*<0.05). Follow-up revealed no significant differences in tumor recurrence and metastasis rates across groups (*p*>0.05).

**Conclusion:**

The modified laparoscopic single-position RNU in combination with bladder cuff resection is a safe and effective minimally invasive approach for UTUC, offering advantages like reduced operative time, early catheter removal, and enhanced patient recovery, supporting its broader clinical application.

## 1. Introduction

Upper tract urothelial carcinoma (UTUC) is a distinct malignancy characterized by multifocality, propensity for metastasis, and an overall unfavorable prognosis.^1^,^2^ Patients diagnosed with UTUC are at a significant risk of recurrence, particularly in the contralateral upper urinary tract following surgical intervention for unilateral disease.^3^,^4^ To achieve effective tumor control, it is crucial to excise the entire urinary tract from the kidney to the bladder’s ureteral orifice.^5^ Consequently, radical nephroureterectomy (RNU) combined with bladder cuff excision (BCE) has become the standard treatment for this condition.

Conventionally, open nephroureterectomy (ONU) involves a flank incision to remove the affected kidney and upper ureter, followed by an incision in the lower abdomen for BCE.^6^ While ONU is a time-honored approach for managing UTUC, it is associated with substantial tissue trauma and a protracted recovery. This technique remains valuable for patients with advanced-stage disease featuring large tumors and extensive local invasion, which may warrant more invasive measures.

In the 1990s, Lin *et al*.^7^ introduced laparoscopic RNU (LRNU), demonstrating the feasibility of minimally invasive techniques in UTUC management.^7^ In recent years, LRNU has gained popularity due to its numerous advantages, including reduced tissue trauma, fewer complications, and shorter recovery times.^8^ Clinical studies comparing ONU with LRNU have consistently demonstrated that LRNU is equally efficacious in controlling tumors, safer, and less invasive.^9^-^12^

Currently, two primary approaches are available for the removal of the affected kidney and upper ureter: Transabdominal and retroperitoneal.^13^ Many urologists favor the retroperitoneal route for LRNU out of anatomical considerations, as it facilitates the access to renal vessels and minimizes disruption to surrounding gastrointestinal organs. This approach promotes the post-operative recovery and lowers the risk of gastrointestinal complications.^13^ However, challenges arise when handling larger tumors or performing the procedures on obese patients, as limited working space can complicate the surgery. Moreover, managing the distal ureter within the confined pelvic space can be technically difficult, often entailing supplementary procedures that extend operative times.

The prevailing practice in UTUC treatment involves laparoscopic nephroureterectomy, followed by an open lower abdominal incision for BCE. While this method outperforms traditional surgeries by minimizing overall trauma and effectively addressing the intravesical segment of the ureter, the need for patient repositioning prolongs both operative and anesthetic times.^14^ This repositioning also heightens the risk of intraoperative complications and may lead to more post-operative pain and higher infection risk, thereby compromising the overall benefits of minimally invasive surgery.

Recent research efforts have been directed toward optimizing trocar placement, surgical routes, and techniques for distal ureter excision. Some studies have explored combining LRNU with transurethral electrosurgical BCE, which necessitates positional changes that can raise safety concerns and the potential for tumor seeding.^15^ Other approaches employing linear cutting staplers for BCE simplify the procedure but pose challenges in achieving precise excisions, potentially leading to complications such as bladder stones.^15^

Despite the advancements in laparoscopic technologies and surgical techniques, effective excision of the distal ureter and bladder wall remains a challenge due to the ureter’s deep pelvic location and proximity to other anatomical structures.^15^ Here, we have developed a complete laparoscopic, single-position technique that integrates nephroureterectomy and BCE. By continuously refining trocar layout and maintaining optimal patient position, we aimed to enhance the efficiency and achieve standardization of this surgical approach. This paper presents our surgical approach, clinical outcomes, and insights into how to address the existing limitations in the management of upper tract urothelial carcinoma (UTUC).

## 2. Methods

### 2.1. Study population

This retrospective study examined the clinical data of 104 patients diagnosed with UTUC who had undergone RNU at our institution between May 2022 and July 2024. The patients were classified into three groups based on the surgical technique utilized: Group A included 39 patients who underwent modified LRNU with BCE, Group B comprised 38 patients who received conventional LRNU with BCE, and Group C involved 27 patients who had LRNU combined with an abdominal oblique incision for BCE.

### 2.2. Inclusion and exclusion criteria

The inclusion criteria included patients who underwent comprehensive pre-operative evaluations, which consisted of echocardiography, pulmonary function tests, chest computed tomography (CT), routine blood tests, liver function tests, and coagulation studies to rule out any contraindications for surgery. In addition, post-operative pathology was needed to confirm a diagnosis of UTUC.

The exclusion criteria involved several factors. Patients with pre-operative renal dysfunction or those who had previously undergone contralateral nephrectomy due to UTUC or renal cancer were excluded. In addition, patients having pre-operative lymphadenopathy or distant metastases requiring lymphadenectomy during surgery were also excluded. Those diagnosed with concurrent bladder tumors through imaging techniques (CT, urography, magnetic resonance urography) or cystoscopy, necessitating transurethral resection of bladder tumors during the procedure, were also excluded. Finally, cases that required conversion from laparoscopic to open surgery were not eligible for inclusion into the study.

### 2.3. Surgical techniques

**Figure 1:**
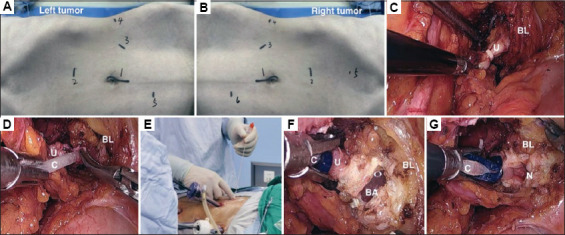
The view outside the operating field and the laparoscopic view of the pre-stitch of the bladder. (A) Location of each trocar pore when the tumor is on the left side. (B) Location of each trocar pore when the tumor is on the right side. (C) Laparoscopic view of the distal ureter end and the sites for closure with a Hem-o-lok clip. (D) Laparoscopic view of the distal ureter opened by scissors and the insertion of a 6F catheter. (E) The view outside the operating field showing the maintenance of the proper tension for the urinary catheter. (F) Laparoscopic view of the bladder and balloon of the catheter. (G) Laparoscopic view of pre-stitching the bladder. Abbreviations: BA: Balloon of the catheter; BL: Urinary bladder; C: Catheter; N: Suture needle; U: Ureter.

**Figure A1:**
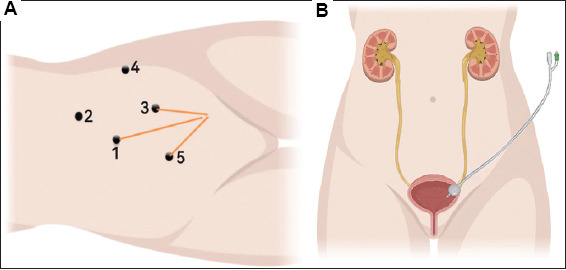
A refined three-dimensional schematic of the key steps for the modified surgical techniques. (A) Location of each trocar pore when the tumor is on the left side. (B) The schematic of the operation field showing the usage of the urinary catheter

For Group A, after successful anesthesia, a triple-lumen catheter was introduced into the bladder for intraoperative irrigation. The patient was positioned in the lateral decubitus position, with a sandbag placed on the back to create a 70° angle with the operating table. A small incision was made near the umbilicus, and pneumoperitoneum was established at a pressure of 10 – 12 mmHg. A 10-mm trocar was inserted, followed by the placement of a laparoscope. Additional trocars, sized 5 mm and 12 mm, were introduced according to tumor laterality. The surgical team used various dissection techniques to carefully detach the kidney and surrounding tissues when avoiding damaging major blood vessels (Figures 1 and A1).

The renal artery and vein were meticulously dissected, clamped with Hem-o-lok ligation clips, and transected using an ultrasonic scalpel prior to tumor manipulation to prevent potential hematogenous dissemination of tumor cells through the renal vasculature. After complete mobilization of the affected kidney and surrounding fat, pirarubicin was infused through the catheter, which was retained for 30 min. If the tumor involved the renal pelvis or proximal ureter, precautions were exercised to prevent spillage of tumor cells during manipulation.

To manage the distal ureter, a 12-mm trocar was inserted approximately 7 cm below the umbilicus, and the surgical approach was adjusted accordingly. A triple-lumen catheter was inserted into the bladder via a small ureteral incision under laparoscopic guidance, ensuring continuous irrigation. Suturing was scrupulously performed to prevent retraction of the bladder wall and ensure secure closure (Figure 1). All groups received a standard 18Fr 3-way silicone Foley catheter, which remained indwelling for 7–14 days.

For Group B, the surgical approach closely resembled that of Group A regarding the management of the kidney and proximal ureter. However, the procedure was specifically adapted to address the distal ureter and bladder wall segment. Surrounding tissues were cleared to expose the connection between the ureter and bladder, and the detrusor muscle was incised to visualize the bladder mucosa. Preliminary sutures were placed to prevent retraction and facilitate closure after BCE was completed.

The primary distinction between Group A and B lies in their approaches to distal ureter and bladder cuff management. Group A utilized a triple-lumen catheter inserted via a small ureteral incision under laparoscopic guidance for continuous irrigation, with careful suturing to prevent bladder wall retraction. While similar in renal/proximal ureter management, the distal approach was modified in Group B by incising the detrusor muscle to expose the bladder mucosa and placing preliminary sutures to facilitate closure after BCE, enhancing precision in bladder wall reconstruction.

In Group C, the patient assumed a lateral decubitus position with the flank elevated. A sterile drape was applied after cleaning the surgical field. An incision was made above the iliac crest, and subsequent layers were dissected to access the retroperitoneal space. A balloon was inflated to expand this space, allowing for direct access to the kidney. As in previous groups, the renal vasculature was meticulously managed with Hem-o-lok clips prior to a complete nephroureterectomy.

After excision, a lower midline incision was made to remove the affected kidney, along with a portion of the ureter. The bladder was inspected for tumors, and any necessary surgical interventions were performed to ensure proper reconstruction. Closure was executed layer by layer, ensuring thorough hemostasis throughout the procedure.

### 2.4. Post-operative monitoring parameters

Data from all cases were systematically recorded, including the following metrics: surgical duration, defined as the total time from skin incision to complete closure; intraoperative blood loss, quantified from various sources such as gauze and suction devices; drain removal timing, indicating the duration until the renal and pelvic drains were removed; catheter removal timing, which reflects the time until the catheter was removed following the healing of the bladder incision; gastrointestinal recovery time, measured as the duration until the first passage of gas post-surgery; and length of hospital stay, defined as the total duration of hospitalization from admission to discharge, based on specific criteria related to patient recovery and stability. Post-operative pathological examination confirmed that all tumors were R0 resections with negative margins.

### 2.5. Follow-up protocol

Post-operative follow-up is essential for monitoring tumor recurrence and metastasis in patients who have undergone surgery for UTUC. Research showed that 80% to 90% of bladder recurrences occur within the first 2 years after UTUC surgery. To minimize the risk of bladder tumor implantation, all patients received immediate intraoperative bladder irrigation with 30 mg of tepoxalin (THP) mixed with 30 mL of sterile saline via a triple-lumen catheter. This solution was allowed to retain in the bladder for 30 min. Patients with bladder recurrence underwent transurethral resection, followed by intensified intravesical chemotherapy. The Bacillus Calmette–Guérin protocol was initiated within 2 – 6 weeks post-transurethral resection, administered as follows: Once a week for six consecutive doses, followed by every 2 weeks for three doses, and subsequently monthly until a year (totaling 19 sessions). If necessary, monthly instillation continued for an additional 1 – 2 years to consolidate therapeutic efficacy. Concurrently, cystoscopy was performed every 3 months to monitor bladder recurrence.

### 2.6. Bladder irrigation and chemotherapeutic regimen

To prevent bladder tumor recurrence, patients were placed on a long-term bladder chemotherapeutic regimen involving THP and Bacillus Calmette–Guérin. The second round bladder irrigation typically commenced a week post-discharge, contingent upon the patient’s recovery status. The initial protocol consisted of weekly irrigations using 30 mg of THP plus 30 mL of sterile saline over a period of eight consecutive weeks. Following this initial phase, the frequency of irrigations was adjusted to once per month for the subsequent year.

### 2.7. Surveillance strategy

The follow-up included regular cystoscopy and imaging examinations to detect potential tumor recurrence. In the 1^st^ year post-surgery, patients were scheduled for cystoscopic examinations every 3 months. From the second to the 3^rd^ year, these evaluations were extended to every 6 months. If no significant abnormalities were identified during these assessments, the follow-up was conducted on annual basis until the end of the 5^th^ year.

In addition to cystoscopy, comprehensive abdominal and pelvic imaging through CT or magnetic resonance imaging (MRI) was performed every 6 months for the first 2 years post-operation. If these imaging studies yielded no meaningful findings, the review frequency was adjusted to annual till the 5^th^ year after surgery. Routine laboratory tests, including complete blood counts, liver function tests, and urinalysis, were conducted during outpatient visits to identify any abnormalities. For patients at high risk of tumor progression, regular chest CT scans and bone scans were performed to assess for distant metastases. Tumor recurrence is defined by the detection of tumors in the bladder or contralateral urinary system by follow-up imaging and cystoscopy.

### 2.8. Definition of tumor metastasis

Tumor metastasis tends to involve lymph nodes at the common sites for UTUC, including the renal hilum, para-aortic region, and areas adjacent to the inferior vena cava. Tumors located in the middle to lower segments of the ureter may also spread to pelvic lymph nodes. This study defined tumor metastasis in terms of the presence of lymph node involvement or distant metastatic disease, as confirmed by post-operative imaging modalities such as CT, MRI, and positron emission tomography-CT.

### 2.9. Pathological staging and grading

Pathological stages post-surgery were determined according to the 2017 edition of the tumor, node, and metastasis (TNM) classification for UTUC. Histopathological grading followed the World Health Organization classification established in 1973, categorizing tumors as G1 (well differentiated), G2 (moderately differentiated), or G3 (poorly differentiated).

### 2.10. Data collection and statistical analysis

Data collection was conducted through outpatient appointment systems, inpatient medical records, and telephone follow-ups. Statistical analyses were performed using the Statistical Package for the Social Sciences version 24.0. Continuous variables were presented as mean ± standard deviation. Comparisons among the three groups of surgical patients, adhering to normal distribution and equal variance, utilized analysis of variance; otherwise, the Kruskal–Wallis test was employed for non-parametric data. Comparisons of categorical data among the groups were made using the Chi-square test. Rates of tumor recurrence and metastasis were compared using Fisher’s exact test. *p*<0.05 was considered statistically significant across all analyses. Subgroup analyses for Group C were considered hypothesis-generating due to limited sample size. The findings were validated in larger cohorts.

## 3. Results

### 3.1. Patient enrollment and demographics

This study included 104 patients, who were divided into three groups based on surgical techniques used: Group A (*n* = 39), Group B (*n* = 38), and Group C (*n* = 27) (Table 1). One case had severe adhesions from prior abdominal surgery (a decision made 45 min into the procedure). Two cases developed uncontrolled bleeding during ureteral dissection (converted at 60 min). These three cases were not included in this study. Demographic comparisons across the groups indicated no statistically significant differences in age, body mass index (BMI), gender, history of hypertension, diabetes status, tumor laterality, presence of hydronephrosis on the affected side, tumor location, and reasons for initial presentation (*p*>0.05). Detailed demographic data are presented in Table 1.

**Table 1:** Baseline characteristics of included participants

Parameters	All patients, *n*=104	Group A, *n*=39	Group B, *n*=38	Group C, *n*=27	*p*-value
Age (year) (mean±SD)	66.4±7.09	67.8±7.46	65.3±5.86	65.8±7.99	0.262
Sex (%)					0.994
Female	38 (36.5)	14 (35.9)	14 (36.8)	10 (37.0)	
Male	66 (63.5)	25 (64.1)	24 (63.2)	17 (63.0)	
Body mass index (kg/m^2^) (mean±SD)	23.7±2.87	23.7±3.05	24.1±2.90	22.9±2.47	0.229
Hypertension (%)					0.572
No	51 (49.0)	21 (53.8)	19 (50.0)	11 (40.7)	
Yes	53 (51.0)	18 (46.2)	19 (50.0)	16 (59.3)
Diabetes (%)					0.572
No	70 (67.3)	25 (64.1)	28 (73.7)	17 (63.0)	
Yes	34 (32.7)	14 (35.9)	10 (26.3)	10 (37.0)
Tumor side (%)					0.848
Left	54 (51.9)	20 (51.3)	21 (55.3)	13 (48.1)	
Right	50 (48.1)	19 (48.7)	17 (44.7)	14 (51.9)
Hydronephrosis (%)					0.907
No	72 (69.2)	26 (66.7)	27 (71.1)	19 (70.4)	
Yes	32 (30.8)	13 (33.3)	11 (28.9)	8 (29.6)
Tumor location (%)					0.089
Lower ureter	37 (35.6)	17 (43.6)	12 (31.6)	8 (29.6)	
Middle ureter	27 (26.0)	10 (25.6)	6 (15.8)	11 (40.7)	
Renal pelvis/upper ureter	40 (38.5)	12 (30.8)	20 (52.6)	8 (29.6)
Symptom (%)					0.763
Hematuria	77 (74.0)	29 (74.4)	30 (78.9)	18 (66.7)	
Lumbago	13 (12.5)	5 (12.8)	3 (7.89)	5 (18.5)	
None	14 (13.5)	5 (12.8)	5 (13.2)	4 (14.8)
TNM stage (%)					0.890
T1	20 (19.2)	8 (20.5)	6 (15.8)	6 (22.2)	
T2	50 (48.1)	19 (48.7)	17 (44.7)	14 (51.9)	
T3	26 (25.0)	8 (20.5)	12 (31.6)	6 (22.2)	
Ta	8 (7.69)	4 (10.3)	3 (7.89)	1 (3.70)
Grade (%)					0.686
G1	9 (8.65)	5 (12.8)	2 (5.26)	2 (7.41)	
G2	56 (53.8)	21 (53.8)	19 (50.0)	16 (59.3)	
G3	39 (37.5)	13 (33.3)	17 (44.7)	9 (33.3)	

Note: Data are presented as n (%), unless otherwise specified. Abbreviations: TNM: Tumor, node, and metastasis; SD: Standard deviation.

### 3.2. Perioperative data comparison

All surgical procedures were successfully completed in each group. No significant differences in drain removal timing were observed among Groups A, B, and C (*p*>0.05; Table 2). However, notable variations were found in several perioperative parameters, including surgical duration, intraoperative blood loss, timing of ureteral catheter removal, gastrointestinal motility recovery time, and length of hospital stay (*p*<0.05; Table 2). Specifically, Group A exhibited significantly shorter surgical times and urinary catheter removal times compared to Groups B and C (*p*<0.01; Table 2). No serious perioperative complications, such as bladder leakage and post-operative massive hemorrhage, occurred in the three groups.

**Table 2:** Comparison of perioperative features in three surgery groups

Parameters	All patients, *n*=104	Group A, *n*=39	Group B, *n*=38	Group C, *n*=27	*p*-value
Operation time (min)	166±29.4	154±27.8	160±26.1	191±20.5	<0.001
Intraoperative blood loss (mL)	42.7±31.5	34.1±17.7	37.6±33.2	62.2±36.9	0.001
Drainage tube removal time (min)	6.11±2.08	5.92±2.16	6.08±2.23	6.41±1.76	0.650
Urinary catheter removal time (day)	11.6±3.93	7.03±1.29	14.0±1.83	14.8±1.84	<0.001
Intestinal recovery (day)	2.91±0.80	2.64±0.67	2.89±0.86	3.33±0.73	0.002
Hospital stay (day)	9.45±2.76	8.87±2.17	9.13±2.86	10.7±3.06	0.016

Note: Data are presented as mean±SD.

### 3.3. Post-operative follow-up outcomes

By the end of the follow-up period in December 2024, which lasted from 5 to 26 months, 14 (13.5%) patients experienced tumor recurrence postoperatively. This included four cases from Group A, six cases from Group B, and four cases from Group C. The rates of tumor recurrence were not significantly different across the groups (*p*>0.05; Table 3). Within Group B, one patient developed a recurrence at the ureteral stump, which was managed through transurethral resection. In Group C, one patient had a recurrence at the contralateral ureteral orifice, which was treated with transurethral laser resection and double-J stent placement. The remaining recurrences were bladder tumors, all requiring surgical resection.

In addition, post-operative imaging (CT or MRI) revealed evidence of distant metastasis in 10 (9.62%) patients: Four in Group A, five in Group B, and one in Group C. The rates of distant metastasis were 10.3% in Group A, 13.2% in Group B, and 3.7% in Group C, showing no significant differences among the groups (*p*>0.05; Table 3). Notably, in Group A, one patient developed pelvic lymph node metastasis 10 months after surgery, while another had liver metastasis at 8 months. In Group B, one patient showed pulmonary metastasis 12 months post-surgery, along with another exhibiting retroperitoneal lymph node and lung metastases at 8 months. Additional metastatic events in Group B included para-aortic lymph node metastasis at 6 months and retroperitoneal lymph node metastasis at 11 months. In Group C, one patient was diagnosed as having bone metastasis 14 months post-surgery. Importantly, the TNM stages were significantly associated with the rate of tumor recurrence and distant metastasis, while the T3 stage had the highest recurrence rate as well as metastasis rate (Table 4).

**Table 3:** Comparison of post-operative follow-up indices by surgery groups

Parameters	All patients, *n*=104 (%)	Group A, *n*=39 (%)	Group B, *n*=38 (%)	Group C, *n*=27 (%)	*p*-value
Recurrence					0.764
No	90 (86.5)	35 (89.7)	32 (84.2)	23 (85.2)	
Yes	14 (13.5)	4 (10.3)	6 (15.8)	4 (14.8)
Metastasis					0.497
No	94 (90.4)	35 (89.7)	33 (86.8)	26 (96.3)	
Yes	10 (9.62)	4 (10.3)	5 (13.2)	1 (3.70)	

Note: Data are presented as *n* (%).

**Table 4:** Comparison of post-operative follow-up indices by tumor, node, and metastasis stages

Parameter	T1, *n*=20 (%)	T2, *n*=50 (%)	T3, *n*=26 (%)	Ta, *n*=8 (%)	*p*-value
Recurrence					0.004
No	20 (100)	45 (90.0)	17 (65.4)	8 (100)	
Yes	0 (0.00)	5 (10.0)	9 (34.6)	0 (0.00)
Metastasis					0.011
No	20 (100)	47 (94.0)	19 (73.1)	8 (100)	
Yes	0 (0.00)	3 (6.00)	7 (26.9)	0 (0.00)	

Note: Data are presented as *n* (%).

Overall, the findings indicated comparable outcomes regarding tumor recurrence and distant metastasis among the three groups, despite variations in perioperative metrics. These results provide valuable insights into the management and outcomes of patients undergoing surgery for UTUC.

## 4. Discussion

Collectively, our data demonstrated that the modified laparoscopic single-position RNU with bladder cuff resection is a safe and effective minimally invasive approach for treating UTUC.^16^ The findings of this study provided valuable insights into the outcomes of patients undergoing surgery for UTUC. While surgical procedures were generally successful, significant differences in perioperative metrics and post-operative outcomes were observed among the treatment groups. These results enhance our understanding of UTUC management and highlight the complexities involved in patient recovery and follow-up.

Demographic data across the three groups revealed no statistically significant differences in key variables such as age, BMI, gender, comorbidities, tumor laterality, and reasons for presentation. This consistency strengthens the validity of the comparisons made within the study, suggesting that observed differences in perioperative and post-operative outcomes can be attributed to the surgical techniques or protocols used rather than inherent patient characteristics. Further analysis demonstrated significant differences in several perioperative parameters, including surgical duration, intraoperative blood loss, ureteral stent removal time, gastrointestinal recovery, and length of hospital stay. Moreover, serious perioperative complications were not observed in all three groups. Notably, the modified surgery exhibited shorter surgical and catheter removal time compared to the other groups. These variations may reflect differences in surgical techniques employed or the experience level of the surgical teams. Shorter surgical durations are often associated with reduced complications and better overall outcomes, aligning with other studies emphasizing the importance of efficiency in oncological procedures. In addition, the absence of significant differences in drain removal timing across groups indicates a standardized approach to post-operative care, which is essential for minimizing complications and promoting recovery. However, the observed variations in other metrics suggest a need for continued refinement of surgical techniques and post-operative protocols to optimize patient outcomes.

A critical aspect of this study was the evaluation of tumor recurrence rates. The overall recurrence rate of 13.5% aligns with existing literature, indicating that the risk of recurrence remains a significant concern following UTUC surgery.^17^,^18^ Importantly, there were no statistically significant differences in recurrence rates among the groups, suggesting that different treatment protocols did not adversely affect the likelihood of tumor return. It is noteworthy that the recurrence rate in the modified group was 10.3%, which was significantly lower than the rates of 15.8% and 14.8% observed in the other groups. However, due to the limited sample size, this apparent advantage in recurrence rate has not achieved statistical significance, indicating the need for further validation in larger studies. Moreover, the relatively low recurrence rates observed in this study underscore the efficacy of prophylactic measures implemented, including immediate post-operative bladder irrigation with THP and long-term chemotherapy with Bacillus Calmette–Guérin and THP. Such interventions are critical to reducing the risk of bladder cancer recurrence, particularly in patients with a history of UTUC. While our follow-up captured critical short-term outcomes, the limited duration precludes definitive conclusions about recurrence patterns beyond 2 years, particularly for indolent variants. Future studies with prolonged surveillance are warranted.

The distant metastasis rate of 9.62% observed in this cohort is consistent with prior studies, reinforcing the notion that while local control is vital, ongoing surveillance for distant spread remains essential.^19^-^21^ The lack of significant differences in metastasis rates among the groups further highlights the effectiveness of the surgical strategies employed. The reported cases of distant metastases, including lymph node, liver, lung, and bone involvement, illustrate the aggressive nature of UTUC and the necessity of vigilant long-term follow-up. While managing local recurrences may involve surgical intervention, distant metastases typically require systemic therapy, presenting additional challenges in treatment options and prognostication.^22^ Comprehensive follow-up strategies, including imaging and laboratory tests, are necessary for identifying these metastases early and manage them effectively.

Above all, the results of this study have important implications for clinical practice. The comparable outcomes with regard to recurrence and metastasis across groups suggest that standardizing surgical approaches and post-operative care protocols may lead to improved patient outcomes without compromising safety. Enhanced communication between surgical teams and urologists regarding follow-up care is also crucial for monitoring and addressing potential complications. Furthermore, these findings reinforce the importance of individualized treatment plans based on patient-specific factors when adhering to established guidelines for managing UTUC.^18^,^23^ Future research should continue to explore risk factors for recurrence and metastasis in UTUC to refine risk stratification and improve therapeutic strategies.

Despite the valuable insights gained from this study, certain limitations must be acknowledged. The retrospective nature of this study limits causal inference. Even with adjustments, unmeasured variables (*e.g*., surgical team expertise) may have influenced outcomes. While the sample size is sufficient for preliminary analyses, it may limit the generalizability of the findings. In addition, the retrospective study introduces potential biases related to data collection and patient selection, including the lack of randomization and blinding. Moreover, the lack of standardized documentation for post-operative systemic therapies may constitute a confounding factor. Based on our institutional protocol, only high-risk patients received adjuvant chemotherapy, while immunotherapy has not yet been incorporated into routine post-operative management for UTUC. Future prospective studies with larger cohorts are needed to validate these findings and explore the long-term outcomes of various surgical techniques and adjuvant therapies in greater depth.

## 5. Conclusion

This study comprehensively analyzed the outcomes associated with the surgical management of UTUC. Although the rates of recurrence and metastasis were similar across groups, the differences in perioperative metrics underscore the importance of refining surgical techniques and post-operative care. Ongoing efforts to understand the complexity of UTUC will ultimately enhance treatment strategies and improve patient outcomes in this challenging area of urology.

## Data Availability

Data will be made available upon reasonable request to the corresponding author.
